# Housing First: exploring participants’ early support needs

**DOI:** 10.1186/1472-6963-14-167

**Published:** 2014-04-13

**Authors:** Vicky Stergiopoulos, Agnes Gozdzik, Patricia O’Campo, Alixandra R Holtby, Jeyagobi Jeyaratnam, Sam Tsemberis

**Affiliations:** 1 Centre for Research on Inner City Health, The Keenan Research Centre in the Li Ka Shing Knowledge Institute of St. Michael’s Hospital, 30 Bond Street, Toronto, Ontario M5B 1W8, Canada; 2 Department of Psychiatry, University of Toronto, 250 College Street, 8th floor, Toronto, Ontario M5T 1R8, Canada; 3 Dalla Lana School of Public Health, University of Toronto, Health Sciences Building, 6th floor, 155 College Street, Toronto, Ontario M5T 3M7, Canada; 4 Pathways to Housing, Inc, New York, NY, USA

## Abstract

**Background:**

Housing First has become a popular treatment model for homeless adults with mental illness, yet little is known about program participants’ early experiences or trajectories. This study used a mixed methods design to examine participant changes in selected domains 6 months after enrolment in a Canadian field trial of Housing First.

**Methods:**

The study sample included 301 participants receiving the Housing First intervention at the Toronto site of the At Home/Chez Soi project. This study used a pre-post design to compare quantitative 6-month outcome data to baseline values in key domains and multivariate regression to identify baseline demographic, clinical or service use variables associated with observed changes in these domains. In addition, qualitative data exploring participant and service provider perspectives and experiences was collected via stakeholder interviews and focus groups, and analyzed using thematic analysis.

**Results:**

The majority (60 to 72%) of participants followed the expected trajectory of improvement, with the remaining experiencing difficulties in community integration, mental health symptom severity, substance use, community functioning and quality of life 6 months after program enrolment. Diagnosis of psychotic disorder was associated with a reduction in quality of life from baseline to 6-months, while substance use disorders were associated with reduced mental illness symptoms and substance use related problems and an improvement in quality of life. Participants housed in independent housing at 6-months had greater improvements in community integration and quality of life, and greater reduction in mental illness symptoms, compared to those not independently housed. The quality of the working alliance was positively associated with improvements in physical and psychological community integration and quality of life. Qualitative data provided a unique window into the loneliness and isolation experienced by Housing First participants, as well as problems related to substance use and a need for life skills training and support.

**Conclusions:**

Additional strategies can help support Housing First participants in the early stages of program participation and address potential causes of early difficulties, including lack of life skills and social isolation. This study highlights the importance of early and ongoing evaluation, monitoring and program adaptations to address consumer support needs.

**Trial registration:**

Current Controlled Trials ISRCTN42520374

## Background

Homelessness is an ongoing social and economic problem that affects thousands of Canadians. In 2009, there were approximately 500 shelters, with a total of more than 17,000 beds, serving homeless individuals and families across Canada [1,2]. In Toronto, Canada’s most populous city [3], more than 5,000 individuals are homeless on any given night [4] and in 2008 approximately 28,000 unique individuals used homeless shelters over the course of the year [5].

Interventions for homeless individuals with mental illness have traditionally focused on a treatment first approach, in which program participants typically progress in a stepwise fashion from emergency shelters to transitional housing before they access permanent supportive housing, often after meeting strict requirements of sobriety and acceptance of psychiatric treatment [6,7]. More recently, Housing First (HF), developed by Pathways to Housing, has emerged as a popular treatment option for meeting the unique needs of this population [8-10]. Rooted in the belief that housing is a basic human right, HF provides individuals with immediate housing, client choice is emphasized in every aspect of treatment, housing is separated from treatment, and a harm reduction approach is followed [8,10].

Previous studies on HF and related programs demonstrate that within one or two years after program entry, a majority of participants experience significant improvements in housing stability [11-13], mental health functioning [14], consumer choice [11], quality of life [13,15] and reductions in health service (emergency and inpatient) use [13], as well as self-reported justice system use [13]. Although reductions in alcohol use have been reported by one study [16], others have found no improvements in either substance or alcohol use after program enrollment [11,17]. In addition to improved participant outcomes, several studies also report on the reduced costs of HF in comparison to traditional housing programs [11,13,14,16,18-20], although some have questioned these cost-savings [21].

Although HF has become a popular treatment option for homeless adults with mental illness, to date the program has only been assessed by a few randomized controlled studies and has not been widely evaluated outside the United States [11,12,20,22,23]. Funded by the federal government through the Mental Health Commission of Canada (MHCC), At Home/Chez Soi (AH/CS) is a 4-year, 5-site demonstration project evaluating the Pathways to Housing HF model and its adaptations in urban and rural settings [23]. The project aims to assess the effectiveness and cost-effectiveness of HF in the Canadian context, and describe the key ingredients necessary for the program’s success and the programs’ theory of change.

### Study goals

**Figure 1:**
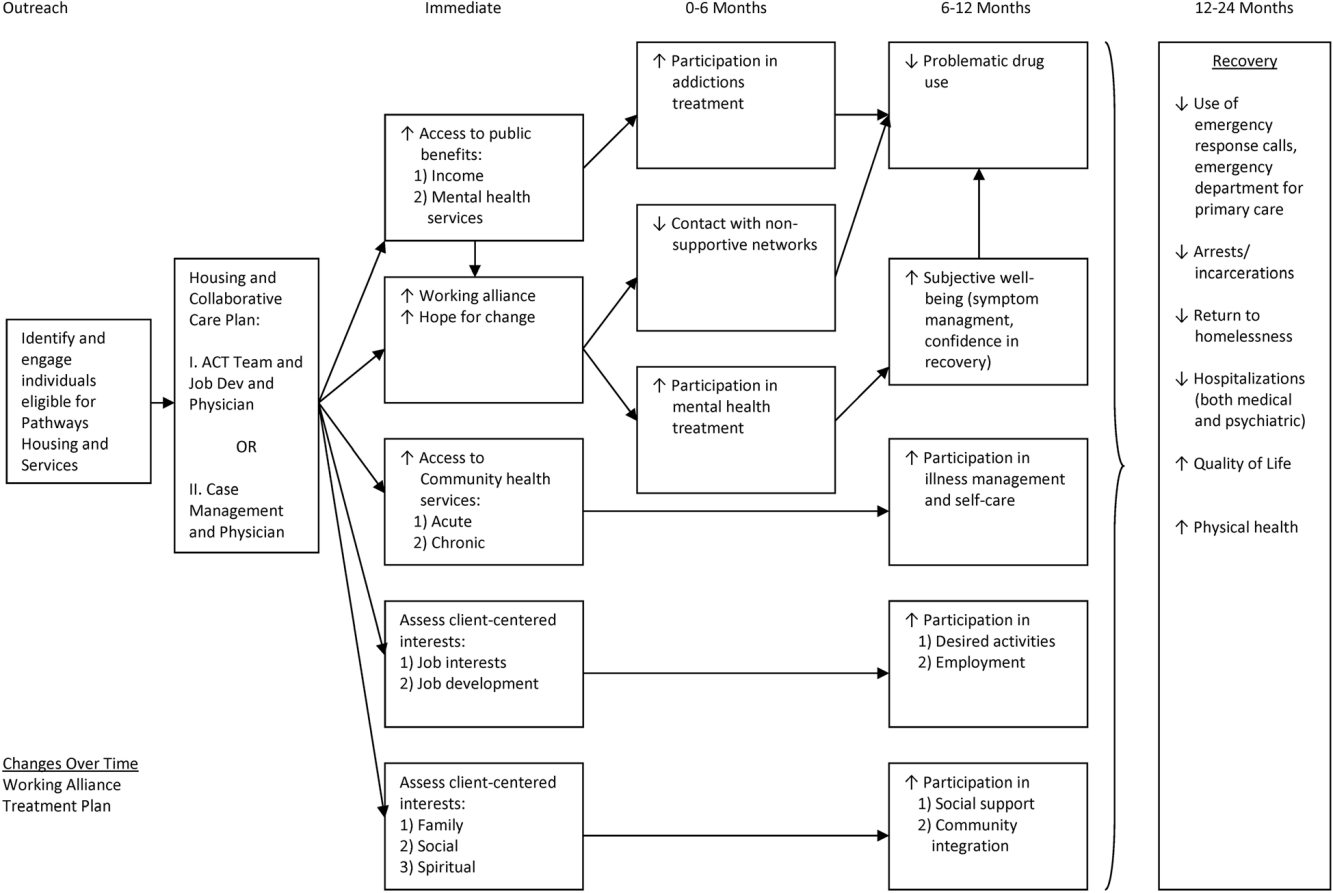
**The At Home/Chez Soi Housing First Program Logic Model (based on Pathways to Housing) [**[10]**].**

Pathways to Housing HF program theory suggest that individuals will experience improvement in several domains over the first and second year of program participation (Figure 1) [10,24]. Despite the growing literature on longer-term outcomes of HF, little has been documented about participants’ early experiences or trajectories [25,26]. Furthermore, the literature on the small number of participants who do not benefit from HF, a target population for alternative interventions and supports, is scant. In response to these knowledge gaps this study uses a mixed-method design to address the following research questions:

1. What proportion of HF participants follow expected trajectories of change in physical and psychological community integration, mental health symptomatology, substance use, community functioning and quality of life 6 months after program enrolment?

2. What baseline demographic, clinical or service use variables are associated with changes from baseline to 6-months in these domains?

3. Are changes from baseline to 6-months in key outcome domains associated with housing and the quality of the relationship between participant and service provider (working alliance)?

4. What are the perspectives of program participants and service providers on early experiences with the program?

These results can guide program planning and resource allocation across jurisdictions striving to improve care and outcomes for homeless adults with mental illness.

## Methods

This study was approved by the Research Ethics Board of St. Michael’s Hospital and is registered with the International Standard Randomized Control Trial Number Register (ISRCTN42520374).

### At Home/Chez Soi study design

The study design, protocol and recruitment process of the AH/CS project, and the Toronto site specifically, have already been described in detail elsewhere [23,27]. Briefly, AH/CS is a randomized controlled trial testing the effectiveness of HF in 5 sites across Canada. Prior to randomization, all eligible participants were stratified into either a “high needs” or a “moderate needs” service group based on their level of need for mental health services [23]. Participants were classified as having high needs if they met all the following 3 criteria: (1) diagnosis of psychotic disorder or bipolar disorder based on the Mini International Neuropsychiatric Interview 6.0 (MINI, see description below); (2) a Multnomah Community Ability Scale (MCAS, see Table 1) score ≤ 62; and (3) at least one of the following: ≥ 2 hospitalizations for mental illness in the past 5 years, recent arrest or incarceration, or comorbid substance use on the MINI [23]. High needs participants randomized to treatment were offered HF with Assertive Community Treatment (HF-ACT). All other participants were considered moderate needs and those randomized to the intervention group received HF with Intensive Case Management (HF-ICM).

**Table 1:** **Domains of Change from Baseline to 6**-**months and Operationalization of** “**Experiencing Difficulties**” **vs**. “**Expected Trajectories**”^**1**^

**Domain**	**Instrument**	**Details**	**Operationalization of expected trajectories and experiencing difficulties**	**Missing at Baseline**	**Missing at 6 Months**
*Community Integration - Physical*	Community Integration Scale (CIS) - Physical subscale	▪7 item subscale of the full 11-item CIS instrument; examines a person’s physical (community presence) integration in the community	**Expected trajectory:** Movement to a higher count, compared to baseline OR no change from baseline	47 (15.6%)	47 (15.6%)
		▪Responses are summed for a total score, with higher scores indicating greater community integration	**Experiencing difficulty:** Movement to a lower count, compared to baseline		
		▪References: [28-30]			
*Community Integration - Psychological*	Community Integration Scale (CIS) - Psychological subscale	▪4 item subscale of the full 11-item CIS instrument; examines a person’s psychological (sense of belonging) integration in the community	**Expected trajectory:** Movement to a higher quintile, compared to baseline OR no change from baseline	6 (1.99%)	54 (17.9%)
		▪Responses are summed for a total score, with higher scores indicating greater community integration	**Experiencing difficulty:** Movement to a lower quintile, compared to baseline		
		▪References: [28-30]			
*Mental Illness Symptomatology*	modified Colorado Symptom Index (CSI)	▪14-item scale that measures the frequency of symptoms of psychiatric illness in the past month	**Expected trajectory:** Movement to a lower quintile, compared to baseline OR no change from baseline	27 (8.97%)	64 (21.3%)
		▪Sum of all 14 items produces the overall CSI score; higher scores indicate greater psychiatric symptomatology; a score greater than 30 indicates the presence of a probable disorder	**Experiencing difficulty:** Movement to a higher quintile, compared to baseline		
		▪References: [31-34]			
*Substance Use*	Global Appraisal of Individual Need – (GAIN-SS) Substance Problem Subscale	▪5-item subscale with individual items scored on a 4-point scale for a given period of time (lifetime or past year or past month or never)	**Expected trajectory:** Movement to a lower count compared to baseline OR no change from baseline	2 (0.66%)	43 (14.3%)
		▪Number of responses with a particular value is counted (depending on time frame under examination)	**Experiencing difficulty:** Movement to a higher count, compared to baseline		
		▪Higher count values indicating higher substance use symptoms			
		▪References: [35,36]			
*Community Functioning*	Multnomah Community Ability Scale (MCAS)	▪17-item instrument that measures the degree of functional ability	**Expected trajectory:** Movement to a higher quintile, compared to baseline OR no change from baseline	0 (0%)	75 (24.9%)
		▪Total MCAS score is sum of all 17 questions. Categories of ability based on total score:	**Experiencing difficulty:** Movement to a lower quintile, compared to baseline		
		−17 to 47 indicates low level of ability			
		−48 to 62 indicates some disability			
		- > 63 indicates little disability			
		▪References: [37-39]			
*Quality of Life*	Quality of Life Inventory (QoLI20)	▪20-item scale that assesses the life circumstances of people with severe and persistent mental illness	**Expected trajectory:** Movement to a higher quintile, compared to baseline OR no change from baseline	26 (8.64%)	71 (23.4%)
		▪A total sum of all items produces a score ranging from 20 to 140, with higher scores indicating greater satisfaction with life	**Experiencing difficulty:** Movement to a lower quintile, compared to baseline		
		▪References: [40-42]			

^1^The last two columns on the right side summarize the extent of the missing data in each of the domains, prior to multiple imputation.

#### Mini International Neuropsychiatric Interview 6.0 (MINI 6.0)

The MINI 6.0 is a short, structured diagnostic interview used for psychiatric evaluation [43] that has been validated against several much longer diagnostic interviews, including the Structured Clinical Interview for DSM Diagnoses (SCID-P) and the Composite International Diagnostic Interview for ICD-10 (CIDI). It shows good concordance and high sensitivity for most diagnosis, with high degree of reliability [43-46].

### Participants

Participants in this study were those randomized to the intervention (HF) arms (HF with ACT or HF with ICM) at the Toronto site of the AH/CS project (N = 301). Briefly, eligibility for the AH/CS project included 1) legal adult age (> 18 years); 2) demonstration of absolute homelessness or being precariously housed; and 3) demonstration of a serious mental disorder with or without a concurrent substance use problem [23]. Participants were excluded if they were currently receiving assertive community treatment (ACT) or intensive case management (ICM), if they were relatively homeless or if they did not have legal status in Canada [23]. Only data collected from the intervention group (HF) of the AH/CS study (N = 301) were utilized for the purpose of this analysis, and participants randomized to the usual care group (N = 274) were excluded. All participants provided written informed consent.

### Quantitative data

This study uses a pre-post design to examine the changes from baseline to 6-months in six outcome domains among participants enrolled in a HF program at the Toronto site of the AH/CS project.

#### Missing data analysis

Missing data in the main outcome domains occurred due to several reasons, including withdrawal, death, loss to follow-up, or participant non-response on specific study instruments, and ranged from 0% to 25% depending on the outcome measured (Table 1). We pursued multiple imputation because complete case analysis (after case deletion) can lead to bias when the data is not missing completely at random (MCAR) [47]. Multiple imputation procedures can improve the plausibility of the missing at random (MAR) assumption when a greater number of observed variables that account for, or are associated with, the reason for missing-ness are incorporated into the model [48,49]. We implemented multiple imputation using the sequential regression multivariate imputation approach (SRMI), also referred to as Fully Conditional Specification (FCS) and Multiple Imputation by Chained Equations (MICE): this method allows for efficient imputation by fitting a model to each variable, conditional on all others, and imputing one variable at a time [50,51]. The multiple imputation model included 1) outcome variables collected at baseline, 6 months, and 12 months); 2) study site; 3) age at enrollment; 4) gender; 5) ethno-racial status and 5) Aboriginal status. Imputed values were restricted to the theoretical range of the original variables by use of bounds. Twenty imputations were stratified by treatment arm and site. Imputations were implemented using IVEware software (http://www.isr.umich.edu/src/smp/ive/), and imputation results were combined using PROC MIANALYZE (SAS 9.3, SAS Institute Inc., Cary, NC).

#### Statistical analyses

All statistical analyses were conducted using IBM SPSS Statistics, version 21 (IBM Corporation, Chicago, IL). A p-value < 0.05 was considered statistically significant.

#### I. Describing early trajectories

To assess improvement in health and social functioning during the early stages of the HF intervention, we examined changes from baseline to 6-months in the following domains:

i) **physical community integration** using the Community Integration Scale (CIS-physical subscale);

ii) **psychological community integration** using the Community Integration Scale (CIS-psychological subscale);

iii) **mental health symptom severity** using the modified Colorado Symptom Index (CSI);

iv) **substance use** problems using the Global Assessment of Individual Need – Short Screener (GAIN-SS) substance use subscale;

v) **community functioning** using the Multnomah Community Ability Scale (MCAS);

vi) **quality of life** using Quality of Life Interview 20 (QoLI20).

See Table 1 for further details on the domains and their associated instruments.

For four of the domains for which the total scale scores were normally distributed (psychological community integration, mental illness symptomatology, community functioning and quality of life), movement between quintiles was used to evaluate trajectories from baseline to 6 months. For each scale examined, participants who moved from their reference baseline quintile to a quintile indicating lower functioning at the 6-month visit were classified as experiencing difficulties. All participants who remained in the same quintile or moved to a quintile indicative of improved functioning were grouped into the expected trajectory group (Table 1).

Two of the domains we examined used scales for which counts, rather than total scores were calculated (physical community integration and substance use). The physical community integration instrument asks if the participant has engaged in seven specific activities in the community in the past month, and the total count corresponds to the number of “Yes” answers. In the GAIN instrument, the counts correspond to how many times a participant has answered “past month” to a series of five questions describing specific problems related to substance use. For both these instruments, change in counts from baseline to 6 months was used to assess differences between the two time points, rather than movement between quintiles (Table 1).

#### Factors associated with early trajectories

We used multivariate regression to assess the relationship between demographic, clinical and service-use variables with changes from baseline to 6-months in each of the outcome domains. In total, ten variables representing select demographic, clinical, or service use domains collected at baseline were examined in each regression model. Demographic variables included: age (years), sex (male or female), years of school completed, ethnicity (ethno-racial or not) and total length of homelessness (years). Clinical variables included the presence of psychosis or an alcohol or substance abuse or dependence diagnosis based on the MINI International Neuropsychiatric Interview (MINI, described above). Finally, service use variables included the type of support service team participants were assigned to at baseline (ACT or ICM), and the self-reported number of emergency department visits in the 6-months prior to baseline. The residuals from multivariate regression analyses for all outcome domains were checked for normality.

#### Associations with housing and participant-reported working alliance

We also examined if the length of time to access housing, housing status and participant’s relationship with their case manager (working alliance) at 6-months were associated with changes from baseline in each of the outcome domains.

Length of time to first being housed (number of days from program assignment to move-in day) was collected by the support service provider agencies. We first performed correlation analysis to examine if the number of days to first being housed was associated with the degree of change from baseline to 6-months in each of the outcome domains, and secondly used t-tests to examine if the mean changes from baseline to 6-months differed among participants who took longer than average to be housed compared to those who were housed in less than or the average length of time.

Furthermore, housing status was derived from the Residential Time Line Follow Back instrument [23,52]. Participants were asked for their current residence at the time of their 6-month interview. Based on this data, we created a dichotomized variable that identified those who were stably housed in independent housing (own apartment, house or home) from those who were living in any other type of housing. Stable independent housing is a goal of Housing First and is often cited as the preferred housing option among individuals experiencing homelessness and/or mental illness [53-55]. We performed t-tests to examine if the amplitude of change from baseline to six-months differed between those who attained independent housing at 6-months compared to those who had not, in each of the six outcome domains.

Finally, the participant-rated working alliance was based on the summary score of the Working Alliance Inventory–Participant Short Form (WAI-PAR) questionnaire [56,57]. The WAI-PAR consists of a total of 12 questions, which ask the participant what they think and feel about the relationship with their service provider, including with respect to therapy goals and tasks. A total score is tabulated, with a greater score indicating a stronger alliance or agreement between the participant and their service provider. We performed correlation analysis to examine if the WAI-PAR score was associated with the degree of change from baseline to 6-months in each of the outcome domains.

### Qualitative data and analysis

Qualitative data for this study were collected as part of the Toronto site’s early Implementation Evaluation as well as consumer narrative interviews with a subset of study participants.

#### Implementation evaluation

Interviews with key informants and focus groups with service providers and program participants were conducted between December 2010 and January 2011. One research team member, who was not involved in the project implementation, conducted all interviews and focus groups. Both key informants and focus group members were selected in consultation with the AH/CS site governance team and the site’s principle investigators, based on their knowledge of the local implementation process and their integral role in the project.

In total, nine key informants were interviewed, including: the Toronto site coordinator, one principal investigator, three support services team leaders (one from each of the support service teams) and four agency directors (one from each of the support service agencies and the housing agency director).

Seven focus groups were conducted with a total of 44 participants: three focus groups were held with the support team case managers (n = 18); one with the housing tem members (n = 4), and three with consumer participants (n = 22).

All key informant interviews and focus groups participants provided written informed consent. All audio-recordings were transcribed and data was analyzed by a three-member research team comprised of the interviewer, a research coordinator and a study principal investigator. First, transcripts were coded independently by the study interviewer and the principal investigator, and compared for consistency. Once consensus was achieved, the interviewer proceeded to code the remainder of the transcripts. The qualitative team would meet to discuss the codes, their resulting higher-order themes and to condense/consolidate the number of themes emerging from the data. All transcripts were analyzed using NVIVO 9.2 software.

#### Consumer narratives

A sampling strategy was implemented where every 10^th^ participant randomized to the treatment arm was approached for participation in the consumer narratives. This approach was employed to achieve a representative sample. A total of 84 participants from the intervention arm were sampled, 57 were contacted and 36 were interviewed. Interviews took place between March 2010 and June 2011. All participants provided written informed consent. The research team consisted of three research staff with training in conducting in-depth interviews. The interviews were semi-structured, and participants were questioned on their history of homelessness and mental health problems, in addition to daily activities, experiences with mental health and social services, and hopes for the future.

Analysts double coded six interview transcripts, compared the reliability of their codes, and met regularly to compare accuracy of codes and to address discrepancies. Discrepancies in coding were discussed and resolved in consultation with the research team. For more information please see [58].

#### Analytic approach

For the purposes of this manuscript, transcripts from the sources described above were analyzed using thematic analysis [59]. Thematic analysis in analytic approach that identifies, reviews and defines the themes or patterns found in the dataset, by searching across the data for repeated patterns of meaning [59]. Of particularly interest to this study were themes relating to participant experiences during the transition from homelessness to becoming a Housing First participant.

## Results

**Figure 2:**
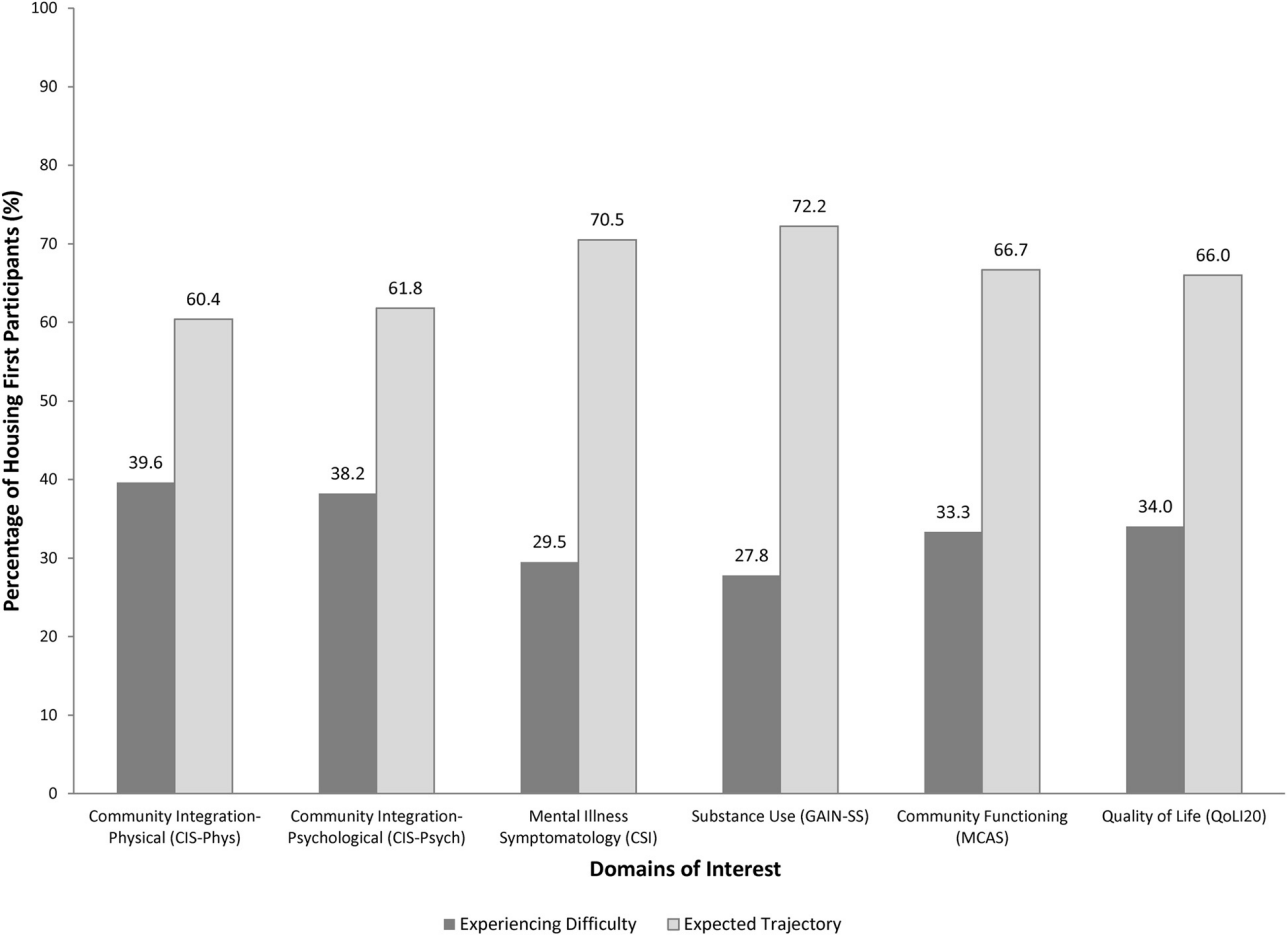
**Housing First participant status at 6 months compared to baseline in key outcome domains (N = 299).** Percentages of participants experiencing difficulties and expected trajectories are based on pooled estimates from 20 imputed datasets.

In total, imputed quantitative data were available for 299 participants both at baseline and 6-months (2 participants had passed away since the baseline interview). We first present changes in the domains of physical and psychological community integration, mental health symptoms, substance use, community functioning and quality of life (Figure 2 and Additional file 1: Table S1), as well as baseline predictors of these changes (Table 2) and their associations with housing status and working alliance at 6-months (Additional file 2: Table S2 and Additional file 3: Table S3). Qualitative data exploring early experiences from the perspective of participants and service providers follows the quantitative findings.

**Table 2:** **Multivariate regression examining the association between participant baseline factors and changes from baseline to 6-months in each of the outcome domains**^**1,2,3**^

	**Community Integration-Physical**				**Community Integration-Psychological**				**Mental Illness Symptomatology**				**Substance Use**				**Community Functioning**				**Quality of Life**			
	_*B*_	_*S.E.*_	_*t*_	_*sig*_	_*B*_	_*S.E.*_	_*t*_	_*sig*_	_*B*_	_*S.E.*_	_*t*_	_*sig*_	_*B*_	_*S.E.*_	_*t*_	_*sig*_	_*B*_	_*S.E.*_	_*t*_	_*sig*_	_*B*_	_*S.E.*_	_*t*_	_*sig*_
Constant	−0.59	0.62	−0.95	0.345	2.00	1.42	1.41	0.160	−4.01	3.34	−1.20	0.231	0.16	0.55	0.29	0.773	1.24	2.39	0.52	0.605	2.02	7.28	0.28	0.782
**Demographic Variables**																								
Age, years	0.01	0.01	0.64	0.522	0.01	0.03	0.18	0.858	0.00	0.06	−0.02	0.982	0.00	0.01	0.23	0.822	0.01	0.04	0.27	0.785	0.17	0.13	1.33	0.185
Gender	0.13	0.27	0.47	0.641	−1.03	0.61	−1.70	0.09	1.59	1.33	1.19	0.232	0.42	0.22	1.89	0.058	−0.60	0.92	−0.65	0.519	1.12	3.03	0.37	0.711
Years of School	0.00	0.00	−0.53	0.599	0.01	0.01	1.04	0.298	0.00	0.01	−0.09	0.926	0.00	0.00	−0.05	0.959	0.01	0.01	1.35	0.178	0.01	0.03	0.53	0.595
Ethnicity	0.25	0.26	0.96	0.338	−0.50	0.62	−0.80	0.422	−0.83	1.40	−0.59	0.553	−0.27	0.24	−1.15	0.250	−0.02	0.98	−0.02	0.986	3.05	3.10	0.98	0.326
Total Years of Homelessness	0.01	0.01	0.99	0.325	−0.04	0.03	−1.61	0.108	0.06	0.05	1.11	0.269	0.00	0.01	0.27	0.791	0.00	0.04	−0.11	0.911	−0.15	0.12	−1.17	0.243
**Clinical Variables**																								
Psychotic Disorder	−0.24	0.27	−0.88	0.381	−0.94	0.61	−1.52	0.128	0.54	1.38	0.39	0.696	0.03	0.22	0.13	0.894	−0.44	0.91	−0.48	0.629	−7.76	3.05	−2.54	0.011
Alcohol or Substance Dependence or Abuse	−0.17	0.26	−0.65	0.515	0.61	0.61	1.01	0.314	−3.72	1.33	−2.80	0.005	−0.47	0.22	−2.13	0.034	0.20	0.93	0.21	0.832	7.03	2.98	2.36	0.018
**Service Variables**																								
Level of Support Service	0.28	0.29	0.97	0.333	−0.84	0.68	−1.23	0.219	0.58	1.51	0.38	0.702	−0.12	0.24	−0.51	0.609	4.63	1.08	4.30	<0.001	−3.15	3.18	−0.99	0.321
Number of Emergency Department Visits	−0.01	0.03	−0.32	0.751	0.04	0.07	0.56	0.577	0.07	0.17	0.41	0.683	−0.03	0.03	−1.02	0.307	−0.03	0.16	−0.21	0.838	0.34	0.37	0.92	0.360

^1^The dependent variable was the calculated change from baseline to 6-months for each of the six outcome domains. Values are pooled from 20 multiply imputed datasets.

^2^Final sample size for the regression analyses was (N = 297) due to some missing data for non-imputed variables, including Gender (N = 2) and Years of School (N = 1).

^3^The categorical variables were as coded as follows: Gender (1 = Female, 0 = Male); Ethnicity (1 = ethno-racial ethnicity, 0 = white ethnicity); Psychotic Disorder (1 = Diagnosis present; 0 = Diagnosis absent); Alcohol or Substance Dependence or Abuse (1 = Diagnosis present; 0 = Diagnosis absent), Level of Support Service, after randomization (1 = Assertive Community Treatment, ACT; 0 = Intensive Case Management, ICM). Zero (0) was the reference category for all categorical variables.

### Early trajectories

Although almost two thirds of participants followed the expected trajectory of improvement for physical (60%) and psychological (62%) community integration, which capture the individuals’ physical presence in the community and individual’s sense of belonging to a community, respectively, the remainder experienced a decrease in their physical (40%) and psychological (38%) community integration from baseline to 6 months (Figure 2). In addition, almost a third (30%) of the participants reported increased mental health symptom severity and more than a quarter of participants (28%) experienced increased problems due to substance use from baseline to six-months. Although two thirds of the participants followed the expected trajectory of improvement in community functioning and quality of life (67% and 66%, respectively), a third (33%) experienced a decrease in community functioning and 34% a decrease in their quality of life at the 6-month interview, compared to the baseline.

### Factors associated with “early difficulties”

Additional file 1: Table S1 shows the means at baseline and 6-months for each of the outcome domains, in addition to the changes in these means between these two time-points. Table 2 shows the results of the multivariate regression examining the relationship between participant baseline factors with changes from baseline to 6-months in each of the domains.

#### Demographic variables

None of the baseline demographic variables were associated with the amount of change from baseline to 6-months in any of the outcome domains.

#### Clinical variables

Diagnosis of psychotic disorder was associated with negative changes from baseline to 6-months in the quality of life domain scores (p = 0.011), indicating a worsening of quality of life among participants with this diagnosis, but was not associated with other outcome domains. Diagnosis of alcohol or substance abuse or dependence was associated with a positive change from baseline in quality of life (p = 0.018) (corresponding to an improvement in quality of life from baseline to 6-months), and a negative change in both mental illness symptomatology (p = 0.005) and substance use (p = 0.034) domains, corresponding to a decrease in both mental health symptom severity and problems associated with alcohol and/or substance use from baseline to 6-months.

#### Service use variables

Compared to ICM participants, ACT participants saw a greater positive change from baseline in community functioning (p < 0.001). The number of emergency department visits in the six months prior to study start was not associated with changes from baseline in the six domains examined.

### Early changes and associations with housing and participant-reported working alliance

The mean length of time from the date of program assignment to being first housed (move-in date) was 68.8 ± 79.3 days, for those participants who remained in the program and were successfully housed at least once (n = 283).

The length of time to housing was negatively correlated with changes from baseline to 6-months in both the community functioning (r = −0.162, p = 0.011) and quality of life (r = −0.127, p = 0.042) domains. A positive correlation was observed between the change from baseline to 6-months in the mental illness symptoms domain and length of time to housing (r = 0.144, p = 0.025) (Additional file 3: Table S3). However, if we only examined data for participants who had been housed within 180 days of randomization (approximately 6 months; n = 264), the length of time to housing was not associated with changes from baseline to 6-months in any of the outcome domains.

None of the domains showed a difference in the mean change from baseline to 6-months in t-tests that compared those who took longer than average length of time to be housed (> 69 days) to those who took the average length of time or less (≤ 69 days) (Additional file 2: Table S2).

Compared to participants who were not independently housed at 6-months, participants who had achieved independent housing experienced greater improvement from baseline in psychological community integration (mean change from baseline: −0.82 vs. 1.79, p = 0.001) and quality of life (mean change from baseline: 4.03 vs. 13.70, p = 0.011). Furthermore, these participants experienced a greater reduction in mental illness symptom severity (mean change from baseline: −2.56 vs. -6.26, p = 0.043) (Additional file 2: Table S2).

The participant-rated working alliance score was positively associated with changes from baseline in three of the outcome domains, including physical community integration (r = 0.165, p = 0.020), psychological community integration (r = 0.142 and 0.044) and quality of life (r = 0.164, p = 0.021) (Additional file 3: Table S3).

### Exploring participant and service provider perspectives

The main themes identified by study participants and service providers in the qualitative interviews and focus groups discussing early program experiences include social isolation, substance misuse and life skills training and support.

#### Social isolation

Participant isolation was the most prominent theme discussed by both service providers and participants. Service providers noted that participants’ move to independent housing often resulted in a change from an environment where they were surrounded by people (e.g. in a shelter or on the street) to one where they are on their own, necessitating early focused efforts to build and/or maintain social networks. One service provider noted that for participants to improve, the program had to “*get* [*the participants*] *a home and then give them a reason to get out of it*”.

Some participants described their feelings of isolation in their narrative interviews. One participant who had recently been housed stated:

“*I have friends in the AA program and kind of a few guys on the streets…but I don’t do very much anymore, I have been staying right in my apartment.*”

Service providers noted that some participants seemed to react to their isolation by “*bringing the streets into their homes*” to replicate the activity they were used to. As one service provider commented:

“*For some participants*, *in the first few months of being housed*, *their contact with non*-*supportive networks actually goes up*…*because they are in a unit and the hustle and bustle of life on the street is not there and they are lonely*, *so they bring it into their unit*”.

For other participants, this initial isolation was an impetus to reconnect with former social networks and family. However, service providers noted that trying to build robust social networks for all participants might be unreasonable, as social isolation is common for residents of Toronto who are housed and who are not mentally ill. One key informant noted:

“*A lot of people in Toronto who aren’t homeless or mentally ill are not very well integrated into the community…there’s a lot of social isolation in general. I am not sure if we’re going to achieve better community integration than what the average Torontonian has.*”

#### Substance misuse

Service providers noted that, for some participants, substance use did not improve in the first few months in housing, and for others it actually increased. One service provider noted that

“*It seems like there’s so much almost inner emptiness that [the participants] have to fill so the problematic drug use, which I think in the long run does go down, I think actually it increases often when they first move in.*”

Service providers were also skeptical that the intervention would be able to reduce participant’s substance use over time. One service provider stated that

“*The [outcome] that I am a little bit uncertain about is decreased problematic drug use. I’m not sure how the model actually, whether the model actually achieved that, and I am not sure the literature is as strong to suggest that we should expect it.*”

Some participants, on the other hand, described a strong desire to reduce or eliminate substance use. Some participants noted that being housed enabled them to reduce substance use by decreasing their exposure to drugs/alcohol and allowing them to leave unsupportive environments, and just “be alone”. However, when being alone turned into being lonely, a few participants acknowledged that their substance use increased. As one participant stated,

“*When I was living in a shelter I always had people to talk to*, *so I didn*’*t really drink when I was in the shelter.*”

#### Life skills training and support

Adjusting to a new environment once housed was seen as a factor that could potentially delay participant’s subjective and objective improvement. The need to learn or re-learn basic living skills following many years of living in institutions or on the street was highlighted by service providers. One provider commented on the need to provide extra support to participants to teach these skills:

“*On the street you knew where to get the food, and now you’re in the west end, like you, you would find somebody in their unit sitting going “What the hell am I doing?”….You have to show them where the garbage is…there’s a lot of stuff they had to be taught.*”

One participant, who was waiting for housing at the time of interview, described his fears of moving:

“*You know, if you go from being in a shelter to going… it’s a big change you know?…I just won’t have anyone to talk to, I won’t have anyone to help me you know, like if I need help doing something or I want to talk to somebody, or I just want to hang out with somebody, I’m going to have to leave my house and go somewhere…I just don’t want to fall back into a depression because of that.”*

## Discussion

Previous studies of HF and supported housing interventions for homeless adults with mental illness have demonstrated that a large majority of participants (typically > 80%) are successfully housed and may improve in other outcomes after 1 or more years [11,12,25,26,60-63]. However, the literature on the transition from being homeless to getting housed in a HF program is sparse. Furthermore, little is known about the group of participants that do not benefit from HF. Exploring participant experiences during the early adjustment period in a HF program can help guide future approaches to address challenges during the transition period from being homeless to becoming housed, as well as inform program adaptations and resource allocation in the growing number of jurisdictions adopting HF.

To inform local planning efforts, this study draws from qualitative and quantitative data to highlight early participant and service provider observations with HF as well as examine early experiences among HF participants 6 months after program enrolment. Although based on the HF program model, approximately two thirds of program participants followed the expected trajectories of improvement at 6-months, the remainder experienced difficulties in community integration (physical and psychological), mental health symptoms substance use, community functioning and quality of life.

Neither the demographic nor the service use variables examined were associated with changes from baseline to 6-months in any of the six domains of interest. Participants diagnosed with psychotic disorder at baseline experienced a reduction in the quality of life domain from baseline to 6-months, compared to those who did not have this diagnosis. Interestingly, participants with diagnosis of alcohol or substance abuse or dependence had greater improvement in quality of life and greater reductions in both mental health symptom severity and substance use problems from baseline to 6-months, compared to participants without these diagnoses. These observations are novel and important because to date there is limited evidence that HF programs can improve substance use or related symptoms [11,64]. These observations may have resulted from compromised quality of life at baseline among individuals with a substance use disorder. Housing and the harm reduction philosophy of Housing First may have offered a welcome respite for this group of participants with concurrent disorders, who are typically ill-served by the service systems designed to support them. These observations will need to be further examined when longer-term outcome data from the project is available.

Individuals receiving HF with ACT experienced greater improvements in community functioning from baseline to 6-months, compared to those receiving HF with ICM, suggesting perhaps that the team structure and resource intensity of ACT may have advantages over ICM at the early stages of program enrolment. To circumvent strict ACT admission criteria, which many homeless people with mental illness do not meet, and optimize use of resources, consideration in the future should be given to HF programs adopting flexible models of community support, such as FACT, capable of ACT intensity, but easier to titrate to consumer needs over time [65]. FACT typically has much higher caseloads than ACT, making it an attractive alternative for consideration for both high and moderate needs participants.

It is promising that participants who had achieved stable independent housing by their six-month interview showed greater positive improvements along the psychological community integration, mental health symptom severity, and quality of life domains. Several Canadian studies report that individuals who are homeless and/or have a mental illness prefer to be housed in independent housing rather than congregate settings [55,66,67]. Immediate access to independent housing of their choice and case management support, grounded in a program philosophy of participant empowerment and choice, form the foundation of the HF approach [10], leading to positive housing and health outcomes in a growing number of studies [12,18,25,34,62,63]. Nonetheless, studies have not consistently found that this community placement leads to community integration among program participants [68].

The association of length of time to housing with changes from baseline to 6-months in the outcome domains of interest is more complex. Although a longer time to housing was associated with lower community functioning and quality of life, and increased mental health symptom severity, these associations were not upheld once participants for whom it took longer than 6-months to be housed were excluded. It is therefore likely that any associations between the outcome domains of interest and length of time to housing were driven by a small number of individuals who experienced significant delays in housing (beyond six months). Longer times to housing may have been influenced by both external factors (such as Toronto’s housing market), as well as participant-specific characteristics (participant’s particular choice of neighborhood and/or unit, factors associated with their mental and physical health, etc.). The subject of housing delays and the participants who experienced them in our sample are the topic of another forthcoming paper [69].

The importance of the therapeutic relationship in achieving positive consumer outcomes has been examined extensively in the psychotherapy literature [70,71] and has also been observed in several studies of homeless adults who experience severe mental illness [72-74], although these findings are not universal [75,76]. We observed that those with a stronger working alliance with their case manager were more likely to have improved outcomes in the community integration (both physical and psychological) and in quality of life domains. Similar to our findings, previously chronically homeless adult participants in a supported housing program who rated their therapeutic alliance in the top 75^th^ percentile (at 3 months post study entry) had the highest subjective quality of life and perceived social support, although no association with other key outcomes, including housing, mental health and substance use was found [75].

Our qualitative findings provided insightful observations into the loneliness and isolation experienced by HF participants, which is difficult to capture using quantitative measures alone. Previous studies have also highlighted that some individuals with mental illness living in community settings report feeling isolated, lonely, lack social supports and do not “fit in” [53,77-80] and in comparison to their non-disabled neighbors, experience decreased levels of community integration, particularly social integration [29,81]. Consistent with these findings, some HF participants in this study describe difficulties transitioning into living alone, noting a sense of loss for those they had known in the shelters or hospitals, and difficulty re-learning life skills.

We anticipate that enhancements in service provision, particularly in the areas of life and social skills training, housing and peer support and opportunities to establish positive social networks, may mitigate some of the challenges that some participants experience in the early stages of a HF intervention. As in the general population, social supports provide an important buffer against stressful life events [82,83] and lack of supportive social networks or reduced social functioning in this population can adversely affect both physical and mental health outcomes [84-87]. Similarly, teaching life skills is important for independent living and housing retention among homeless individuals [88-90] and life skills training provides an effective intervention for previously homeless individuals [91-94].

This study brings to light the importance of early program evaluation to identify the challenges participants face in their early adjustment from homelessness to housing and inform interventions and program enhancements to help mitigate them.

A key challenge emerging from our findings is the difficulty in identifying program participants who may require additional services or supports. The lack of clear “predictors” of early difficulties in this study indicates a need to develop new strategies to help identify participants who may experience challenges. Ensuring staff receive adequate training and supervision to identify and address early difficulties may be helpful. Increased attention to the process of participant engagement and the service provider–participant working alliance may also further support the transition process from homelessness to housing and community reintegration.

This study has some limitations. Some of our variables had considerable level of missing-ness at the 6 month interview which we addressed using multiple imputation approaches supported by the literature. Regression to the mean may be a potential cause of some our observations, however, it is important to note that while a longer time frame for the intervention may have helped address this, it would not allow us to investigate how participants fare in the early transitions in a Housing First intervention, which is the focus of this paper. Although all efforts were made to ensure that this sample was representative of the sample of homeless individuals with mental illness residing in Toronto, a small number of individuals who were recruited refused consent to participate in a randomized trial (54 of 726 who were assessed for eligibility declined consent [27]). However, this is a limitation shared with most studies of homeless and/or other vulnerable populations. Another potential limitation is that quantitative and qualitative baseline data for study participants may have been collected on different dates, although the window of capture for the quantitative data would have covered the qualitative data collection period; it is of note, however, that qualitative data include not only the perspectives of program participants captured by our quantitative analyses, but those of their service providers, adding a rich perspective and understanding of the transition from homelessness to housing. While we selected 6-months as our point of focus, it is possible that earlier or later evaluation would highlight additional key elements to participant’s outcomes. Furthermore, this study focused on selected outcomes based on the program’s proposed theory of change. It is possible that examination of other domains would expose other trajectories and relationships.

## Conclusion

This study demonstrates how an early program evaluation can highlight opportunities for program adaptations to better support participants’ trajectories of improvement. Housing First programs should consider strategies to identify participants in need of additional supports early upon program entry, in conjunction with early focused interventions to increase life skills, address substance use and promote social and community integration.

## Competing interests

The authors declare that they have no competing interests.

## Authors’ contributions

VS conceived of the study, interpreted results, contributed to the drafting of the manuscript and read and revised all drafts of the manuscript. AG developed the analysis plan, analyzed the quantitative data, interpreted the findings and drafted the manuscript. PO helped interpret the results and contributed to drafting of the manuscript. AH analyzed the qualitative data and contributed to the drafting of the manuscript. JJ analyzed the qualitative data and contributed to the drafting of the manuscript. ST interpreted the results and contributed to drafting of the manuscript. All authors read and approved the final manuscript.

## Pre-publication history

The pre-publication history for this paper can be accessed here:

http://www.biomedcentral.com/1472-6963/14/167/prepub

## Supplementary Material

Additional file 1: Table S1 Means (standard errors) for each of the domains at both baseline and 6 months, in addition to the mean change from baseline to 6 months^1^. Click here for file

Additional file 2: Table S2 Mean change from baseline to 6-months in outcome domains by length of time to housing and housing status at 6 months^1,2^. Click here for file

Additional file 3: Table S3 Correlation coefficients between changes from baseline to 6-months for each outcome domain and time to housing and the participant-reported Working Alliance total score^1^. Click here for file
