# Supplementary material for: Housing First: exploring participants’ early support needs
Source: BMC Health Serv Res. 2014 Apr 13;14:167. doi: 10.1186/1472-6963-14-167 (PMC4021373; doi:10.1186/1472-6963-14-167)
Supplement: Additional file 1: Table S1 — Means (standard errors) for each of the domains at both baseline and 6 months, in addition to the mean change from baseline to 6 months1. [file 1472-6963-14-167-S1.doc]

**Additional File 1**

**Table S1** Means (standard errors) for each of the domains at both baseline and 6 months, in addition to the mean change from baseline to 6 months^1^

| **Domain** | **Baseline Mean (SE)** | **6-Month Mean (SE)** | **Mean Change (SE)** |
| --- | --- | --- | --- |
| **Community Integration - Physical^2^** | 2.36 (0.10) | 2.20 (0.12) | -0.18 (0.12) |
| **Community Integration - Psychological** | 10.9 (0.21) | 12.1 (0.21) | 1.17 (0.27) |
| **Mental Illness Symptomatology** | 39.72 (0.75) | 34.5 (0.73) | -5.20 (0.63) |
| **Substance Use^3^** | 1.49 (0.11) | 1.39 (0.11) | -0.11 (0.11) |
| **Community Functioning** | 61.68 (0.38) | 64.7 (0.49) | 3.00 (0.47) |
| **Quality of Life** | 73.55 (1.40) | 84.6 (1.30) | 11.14 (1.40) |

^1^ Values are pooled from across 20 multiply imputed datasets.

^2^ Median values: Baseline = 2.00; 6 Months = 2.00; Change = 0

^3^ Median values: Baseline = 0.00; 6 Months = 1.00; Change = 0
