# Supplementary material for: Housing First: exploring participants’ early support needs
Source: BMC Health Serv Res. 2014 Apr 13;14:167. doi: 10.1186/1472-6963-14-167 (PMC4021373; doi:10.1186/1472-6963-14-167)
Supplement: Additional file 2: Table S2 — Mean change from baseline to 6-months in outcome domains by length of time to housing and housing status at 6 months1,2. [file 1472-6963-14-167-S2.doc]

**Additional File 2**

**Table S2** Mean change from baseline to 6-months in outcome domains by length of time to housing and housing status at 6 months ^1,2^

| **Domain** | **Time to Housing^3^** | | | **Housing status at 6 months^4^** | | |
| --- | --- | --- | --- | --- | --- | --- |
|  | Less than or average length of time to be housed (<=69 days) | Longer period of time to be housed (>69 days) | p-value | Independently Housed | Not independently housed | p-value |
| **Community Integration - Physical** | -0.30 | 0.11 | 0.119 | -0.16 | -0.17 | 0.971 |
| **Community Integration - Psychological** | 1.13 | 1.63 | 0.431 | -0.82 | 1.79 | 0.001 |
| **Mental illness Symptomatology** | -5.91 | -3.55 | 0.095 | -2.56 | -6.26 | 0.043 |
| **Substance Use** | -0.14 | -0.02 | 0.572 | -0.28 | -0.08 | 0.457 |
| **Community Functioning** | 3.49 | 2.19 | 0.206 | 1.48 | 3.57 | 0.095 |
| **Quality of Life** | 12.58 | 7.63 | 0.113 | 4.03 | 13.70 | 0.011 |

^1^ Values are pooled from across 20 multiply imputed datasets.

^2^ P-values correspond to results from t-tests comparing mean changes from baseline.

^3^ n=281

^4^ n=264
