# Supplementary material for: Housing First: exploring participants’ early support needs
Source: BMC Health Serv Res. 2014 Apr 13;14:167. doi: 10.1186/1472-6963-14-167 (PMC4021373; doi:10.1186/1472-6963-14-167)
Supplement: Additional file 3: Table S3 — Correlation coefficients between changes from baseline to 6-months for each outcome domain and time to housing and the participant-reported Working Alliance total score1. [file 1472-6963-14-167-S3.docx]

**Additional File 3**

**Table S3** Correlation coefficients between changes from baseline to 6-months for each outcome domain and time to housing and the participant-reported Working Alliance total score ^1^

| **Domain** | **Time to Housing^2^** | | **Working Alliance^3^** | |
| --- | --- | --- | --- | --- |
|  | Correlation Coefficient | p-value | Correlation Coefficient | p-value |
| **Community Integration - Physical** | 0.08 | 0.245 | 0.17 | 0.020 |
| **Community Integration - Psychological** | -0.04 | 0.548 | 0.14 | 0.044 |
| **Mental illness Symptomatology** | 0.14 | 0.025 | -0.06 | 0.432 |
| **Substance Use** | 0.06 | 0.317 | 0.05 | 0.450 |
| **Community Functioning** | -0.16 | 0.011 | 0.07 | 0.384 |
| **Quality of Life** | -0.13 | 0.042 | 0.16 | 0.021 |

^1^ Values are pooled from across 20 multiply imputed datasets.

^2^n=281

^3^n=215
